# Corn-like, recoverable γ-Fe_2_O_3_@SiO_2_@TiO_2_ photocatalyst induced by magnetic dipole interactions

**DOI:** 10.1038/s41598-017-07417-z

**Published:** 2017-07-31

**Authors:** Fang Wang, Manhong Li, Lifang Yu, Fan Sun, Zhuliang Wang, Lifang Zhang, Hao Zeng, Xiaohong Xu

**Affiliations:** 1 School of Chemistry and Materials Science of Shanxi Normal University & Key Laboratory of Magnetic Molecules and Magnetic Information Materials of Ministry of Education, Linfen, 041004 China; 2 Department of Physics, University at Buffalo, SUNY, Buffalo, NY 14260 USA; 3 Research Institute of Materials Science of Shanxi Normal University & Collaborative Innovation Center for Shanxi Advanced Permanent Magnetic Materials and Technology, Linfen, 041004 China

## Abstract

Corn-like, γ-Fe_2_O_3_@SiO_2_@TiO_2_ core/shell heterostructures were synthesized by a modified solvothermal reduction combined with a sol-gel method. SiO_2_ shells were first deposited on monodisperse Fe_3_O_4_ microspheres by a sol-gel method. Fe_3_O_4_@SiO_2_@TiO_2_ corn-like heterostructures were then obtained by sequential TiO_2_ coating, during which the magnetic dipolar interactions induced the anisotropic self-assembly process. After annealing at 350 °C, the crystalized TiO_2_ enhanced photocatalytic activity, while Fe_3_O_4_ was converted to γ-Fe_2_O_3_. The corn-like γ-Fe_2_O_3_@SiO_2_@TiO_2_ photocatalyst can be recycled and reused by magnet extraction. Despite the photocatalytic activity decreased with each cycle, it can be completely recovered by moderate heating at 200 °C.

## Introduction

Much effort has been devoted to environmental protection by degrading pollutants and developing new clean energy sources in recent years. TiO_2_ is an attractive material for such applications due to its excellent electronic and optical properties, and high chemical and thermal stability^1–5^. These features make it useful for photocatalytic decomposition of pollutants^6–8^, dye-sensitized solar cells^9–11^, and photochemical water splitting^4, 5, 12^. Most research focuses on extending the range of its absorption spectrum and thus improving its photocatalytic efficiency^2, 3, 13, 14^, while their recyclability issue has been scarcely addressed. Typically, aqueous suspensions of TiO_2_ nanoparticles employed for most of the photocatalytic reactions are difficult to separate and recycle. Because of this, catalyst immobilization has been proposed to recycle the catalysts. For example, researchers have studied TiO_2_ immobilization over various inactive supports such as glass, quartz and stainless steel substrates^15, 16^. However, the photocatalytic activity would be decreased significantly due to the immobilization, which reduces the active surface area for photocatalysis^17^. Therefore, there is a need to develop a multi-functional photocatalytic system with high photoactivity and recyclability.

Recently, magnetic targeting and magnetic recycling technologies were widely used in biomedical^18–22^ and catalysis fields^23–25^. This is realized by employing functional composite materials with a magnetic component, which make them separable in an external magnetic field. Moreover, the dipole interaction can induce particle aggregation to form chain-like structures^26–30^. Butter *et al*. have directly observed the dipolar chains in iron ferrofluids by cryogenic electron microscopy without applying a magnetic field^26^. Moreover, the dipole-dipole interactions depend on the magnetic particle size and coating molecules^27, 28^. Zhang *et al*. reported that the particle chain length could be effectively adjusted by the intensity of the magnetic field in the range of micrometers and the packing of Fe_3_O_4_ microspheres (~150 nm) in the chains became tighter with increasing field strength^29^. Therefore, an effective and repeatable assembly of magnetic chains is a significant step toward realizing their potential applications spreading from nano-scale electronic devices, sensors and high-density data storage media to controlled drug delivery and cancer diagnostics/treatment systems^30^.

Up to now, many types of magnetic photocatalysts have been synthesized, such as γ-Fe_2_O_3_@SnO_2_^31^, Fe_3_O_4_@TiO_2_@Ag^32^, Fe_3_O_4_@TiO_2_^33, 34^, bean-like core/shell Fe_3_O_4_@C@Cu_2_O^35^. α-Fe_2_O_3_@TiO_2_^36^ and α-Fe_2_O_3_/Ag/SnO_2_^37^ photocatalysts have also been reported. Here the antiferromagnetic α-Fe_2_O_3_ primarily acts as a visible-light photocatalyst due to its narrow band gap (2.2 eV). On the other hand, ferrimagnetic Fe_3_O_4_ and γ-Fe_2_O_3_ with relatively high magnetization are chosen as magnetic cores in magnetically separable photocatalysts^25^. It is reported by Li *et al*. that the direct contact between magnetic Fe_3_O_4_ and TiO_2_ photocatalyst usually results in an increase in electron-hole recombination and photodissolution^35^. In order to overcome the problem of charge recombination, mesoporous TiO_2_/SiO_2_/Fe_2_O_3_^38^, (γ-Fe_2_O_3_@SiO_2_)n@TiO_2_ hybrid nanoparticles with γ-Fe_2_O_3_@SiO_2_ fine particles dispersed in a TiO_2_ matrix^39^, and γ-Fe_2_O_3_@SiO_2_@TiO_2_ composite microspheres with SiO_2_ barrier layers^40^ were synthesized. Compared with Fe_3_O_4_@TiO_2_, the insert of SiO_2_ shell between γ-Fe_2_O_3_ core and TiO_2_ shell exhibits two positive effects to enhance the photocatalytic activity. One is to block the electron injection from TiO_2_ to γ-Fe_2_O_3_ at the interface, the other is to provide a porous surface with large surface-to-volume ratio for catalytic reactions^40^. However, the photogenerated electrons can still transfer if the thickness of the SiO_2_ is less than 5 nm^41^. Therefore, the thickness of SiO_2_ is a key factor responsible for the photocatalytic performance of iron oxide/SiO_2_/semiconductor systems. While most works focus on the recoverability of magnetic core-shell photocatalysts, surprisingly little has been reported on the regeneration and reuse of such photocatalysts.

Here, we report the synthesis and characterization of a corn-like, anisotropic γ-Fe_2_O_3_@SiO_2_@TiO_2_ heterostructure. The formation mechanism of the anisotropic heterostructure is proposed, revealing the importance of magnetostatic interaction as a tuning knob for morphological control. The heterostructure demonstrates photocatalytic activity for degradation of Rhodamine B and can be magnetically recycled and reused, Moreover, the lost photocatalytic activity of the used γ-Fe_2_O_3_@SiO_2_@TiO_2_ heterostructure can be fully recovered by heating at 200 °C for 30 min. Such magnetically recyclable, easily regenerated γ-Fe_2_O_3_@SiO_2_@TiO_2_ composite provides a design paradigm for low cost photocatalysts for renewable energy and environmental applications.

## Results and Discussion

### Structural and morphology characterizations

**Figure 1:**
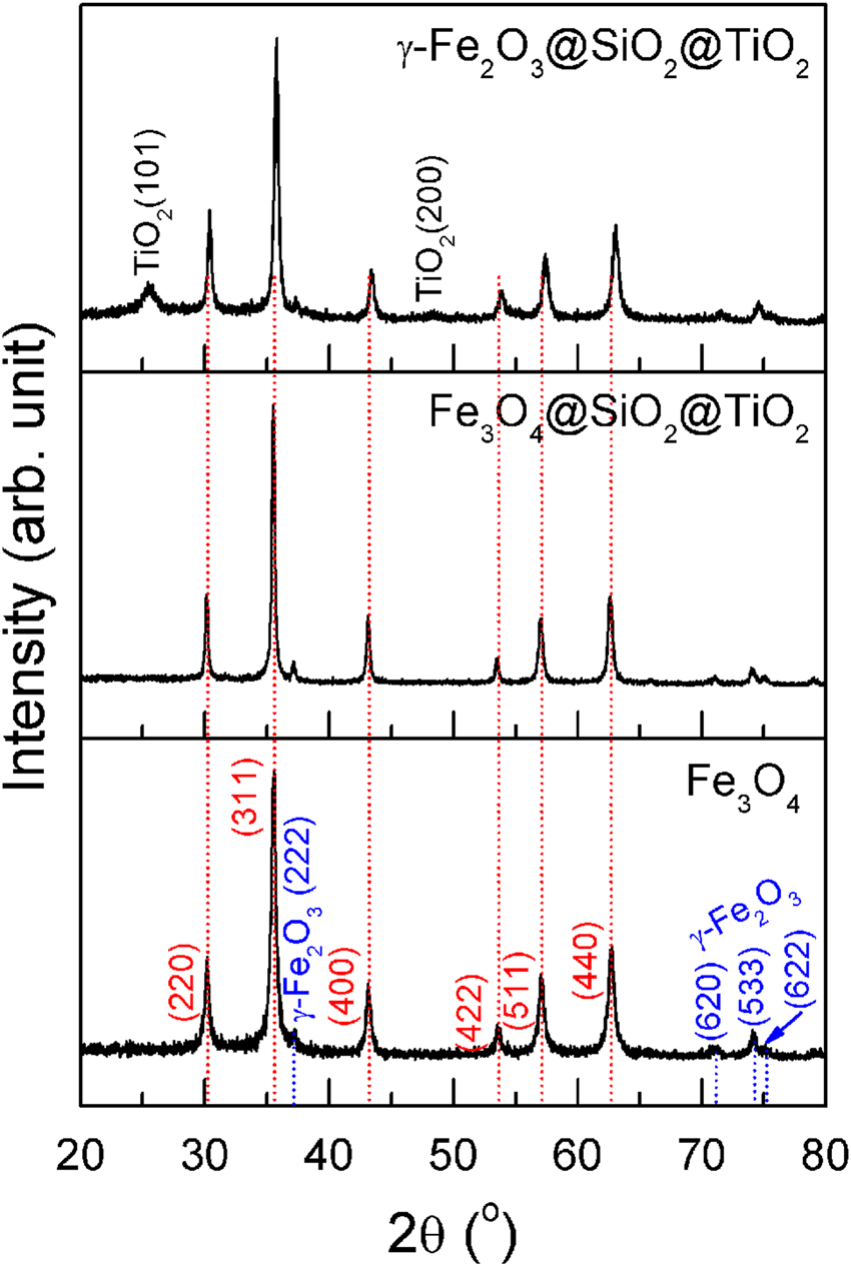
XRD patterns of Fe_3_O_4_, Fe_3_O_4_@SiO_2_@TiO_2_, and γ-Fe_2_O_3_@SiO_2_@TiO_2_ heterostructures.

Figure 1 shows the XRD patterns of Fe_3_O_4_ and Fe_3_O_4_@SiO_2_@TiO_2_ heterostructures before and after annealing. For the Fe_3_O_4_ microspheres, the characteristic XRD peaks of magnetite with inverse spinel structure were observed, indicating that Fe_3_O_4_ phase is obtained by the solvothermal method. However, a minor amount of γ-Fe_2_O_3_ was present in the microspheres. As-synthesized Fe_3_O_4_@SiO_2_@TiO_2_ shows only the similar peaks of Fe_3_O_4_ microspheres without any characteristic peaks of SiO_2_ and TiO_2_. In contrast, when Fe_3_O_4_@SiO_2_@TiO_2_ was annealed at 350 °C for 2 h in air, a clear XRD peak at 25.5° corresponding to the (101) crystal planes of anatase TiO_2_ emerged. Meanwhile, a weak TiO_2_ (200) was also observed after annealing. Thus, TiO_2_ should be an amorphous phase in the as-synthesized Fe_3_O_4_@SiO_2_@TiO_2_ composite. As for SiO_2_, no XRD peaks is seen after annealing at 350 °C for 2 h, suggesting that it remains amorphous despite the heat treatment. However, all the original peaks of Fe_3_O_4_ show a shift to higher 2*θ* values, which correspond to the peaks of γ-Fe_2_O_3_^42^. Moreover, the change in color of the heterostructures from black to brownish-red also suggests the oxidation of Fe_3_O_4_ into γ-Fe_2_O_3_. Thus, γ-Fe_2_O_3_@SiO_2_@TiO_2_ heterostructures can be obtained by such heat treatment in air.

**Figure 2:**
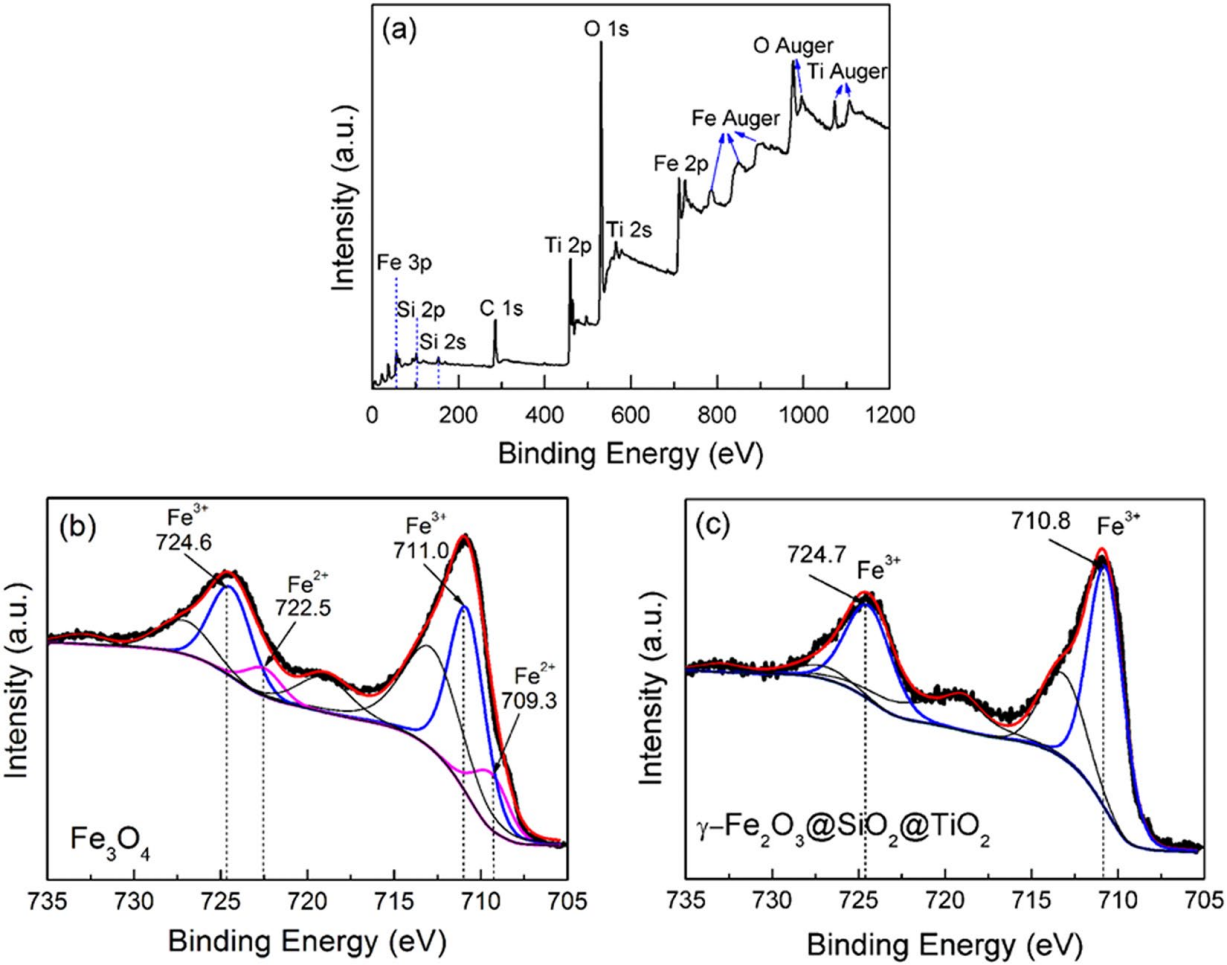
(**a**) A full-scan XPS spectra of γ-Fe_2_O_3_@SiO_2_@TiO_2_ heterostructure, (**b**,**c**) The Fe 2*p* core-level XPS spectra for Fe_3_O_4_ microspheres and γ-Fe_2_O_3_@SiO_2_@TiO_2_ heterostructure.

In order to further confirm the coating of SiO_2_ and oxidation of Fe_3_O_4_, the elemental and valence state analysis were performed using XPS. Here the chemical states of the constituent elements were calibrated by C 1*s* peak (284.6 eV). Figure 2(a) shows the survey scan XPS spectrum of γ-Fe_2_O_3_@SiO_2_@TiO_2_, which contains O 1*s*, Ti 2*p*, Fe 2*p*, Si 2*p* and Si 2*s* peaks. This indicates the existence of SiO_2_ and TiO_2_ in γ-Fe_2_O_3_@SiO_2_@TiO_2_ heterostructure. Figures 2(b) and (c) show the core-level XPS spectra of Fe 2*p* in Fe_3_O_4_ microspheres and γ-Fe_2_O_3_@SiO_2_@TiO_2_ heterostructures, respectively. For the Fe_3_O_4_ in Fig. 2(b), the main-peaks located at 711.0 eV and 724.6 eV are attributed to Fe^3+^ 2*p*3/2 and Fe^3+^ 2*p*1/2, respectively. However, there is a significant asymmetry for both of them. A double peak fitting yields peak positions at 709.3 eV and 722.5 eV, which belong to Fe^2+^ 2*p*3/2 and Fe^2+^ 2*p*1/2, respectively. These results are in agreement with the reported Fe *2p* XPS spectrum of Fe_3_O_4_^43^. In addition, the area ratio of Fe^3+^/Fe^2+^ is about 3.6, which deviates from the stoichiometry of Fe_3_O_4_. Such a deviation indicates that Fe^2+^ ions have been partially oxidized to Fe^3+^ ions, which is in agreement with the XRD results in Fig. 1, which show trace amount of γ-Fe_2_O_3_. Here the FeO_x_ can reflect the coexistence of Fe_3_O_4_ and γ-Fe_2_O_3_. As for the Fe *2p* core-level XPS spectrum of γ-Fe_2_O_3_@SiO_2_@TiO_2_ [Fig. 2(c)], it presents only the peaks of Fe^3+^ at 710.8 eV and 724.7 eV. But, it is hard to observe the peak of Fe^2+^ at about 709.0 eV and 723 eV. Such results suggest that Fe_3_O_4_ in the Fe_3_O_4_@SiO_2_@TiO_2_ heterostructure has been oxidized to γ-Fe_2_O_3_ after annealing at 350 °C for 2 h in air. The difference in color further provides the evidence for oxidation.

**Figure 3:**
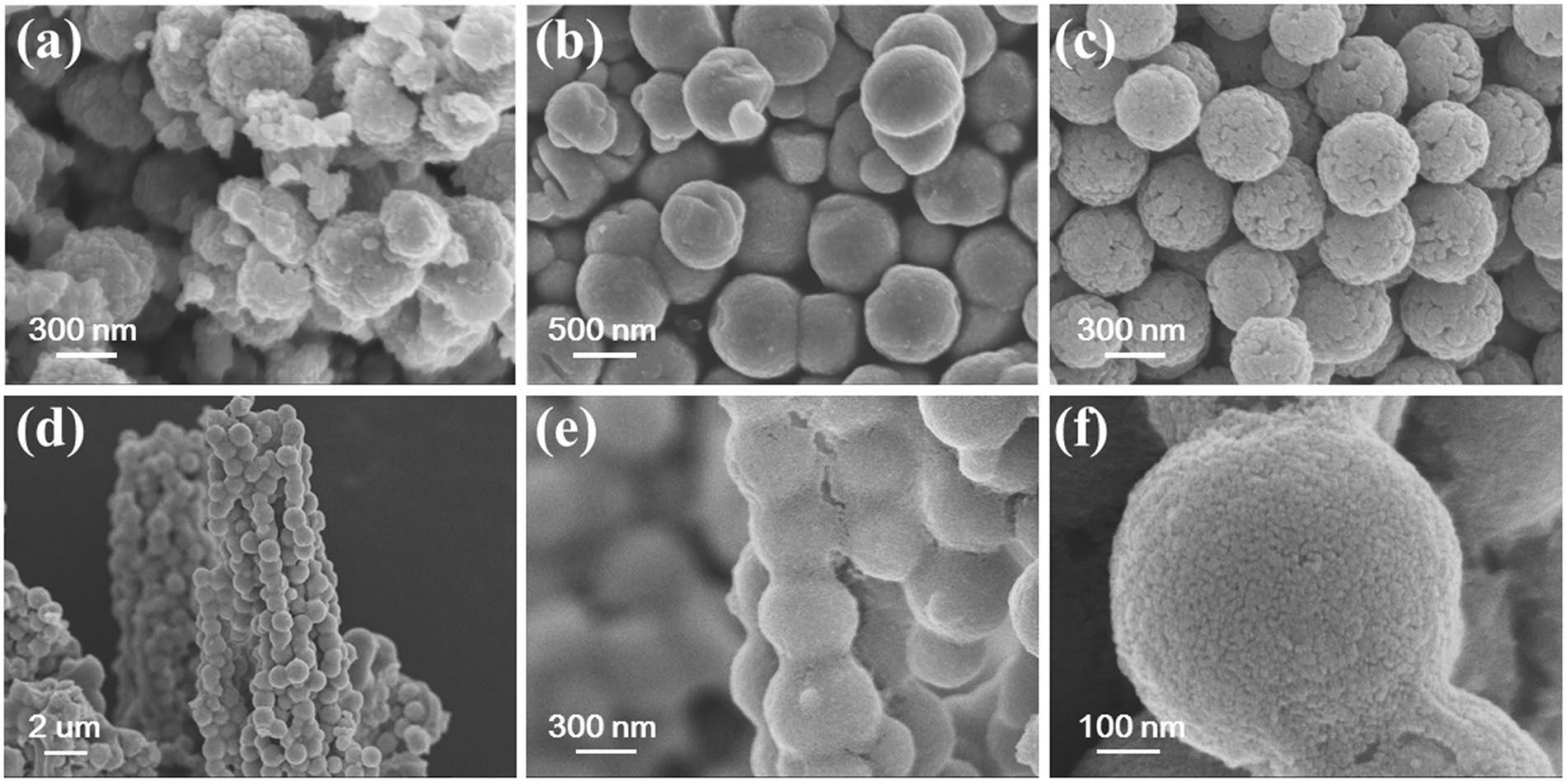
Typical SEM images of Fe_3_O_4_ microspheres synthesized with different surfactants: (**a**) ED, (**b**) PEG, (**c**) NaPAA, and (**d**)–(**f**) γ-Fe_2_O_3_@SiO_2_@TiO_2_ corn-like heterostructures under different magnifications.

Figure 3 shows the SEM images of Fe_3_O_4_ microspheres synthesized by different surfactants and γ-Fe_2_O_3_@SiO_2_@TiO_2_ corn-like heterostructures. As seen from Fig. 3(a)–(c), the surfactants play an important role in controlling the surface morphology of Fe_3_O_4_ microspheres. For the ED surfactant, it can be seen that the spherical morphology is not completely formed, and Fe_3_O_4_ shows some irregular shape [Fig. 3(a)]. When PEG is chosen as the surfactant, Fe_3_O_4_ microspheres with a smooth surface can be obtained [Fig. 3(b)]. On the other hand, as NaPAA is chosen as the surfactant, the Fe_3_O_4_ microspheres demonstrate rough surface. The surface consists of uniformly sized nanoparticles, and the mean diameter of the microspheres is about 450 nm [Fig. 3(c)]. These well-dispersed, rough Fe_3_O_4_ microspheres with large surface-to-volume ratio were chosen as templates for coating the SiO_2_ coupling layer and subsequently the TiO_2_ functional shell. It can be seen from Fig. 3(d)–(f) that γ-Fe_3_O_4_@SiO_2_@TiO_2_ shows anisotropic corn-like structure after TiO_2_ coating. The discrete Fe_3_O_4_ microspheres are now linked by the SiO_2_/TiO_2_ coating to self-assemble into corn-like heterostructures with length exceeding 10 μm. The newly formed TiO_2_ shell is uniformly coated onto the magnetic Fe_3_O_4_ core, leading to a smoother surface than that of the starting Fe_3_O_4_ microspheres. Interestingly, this corn-like heterostructure is only found after coating with TiO_2_ shell, while not observed in the Fe_3_O_4_@SiO_2_ composite. Therefore, the TiO_2_ shell should play a crucial role in forming the corn-like heterostructure.

**Figure 4:**
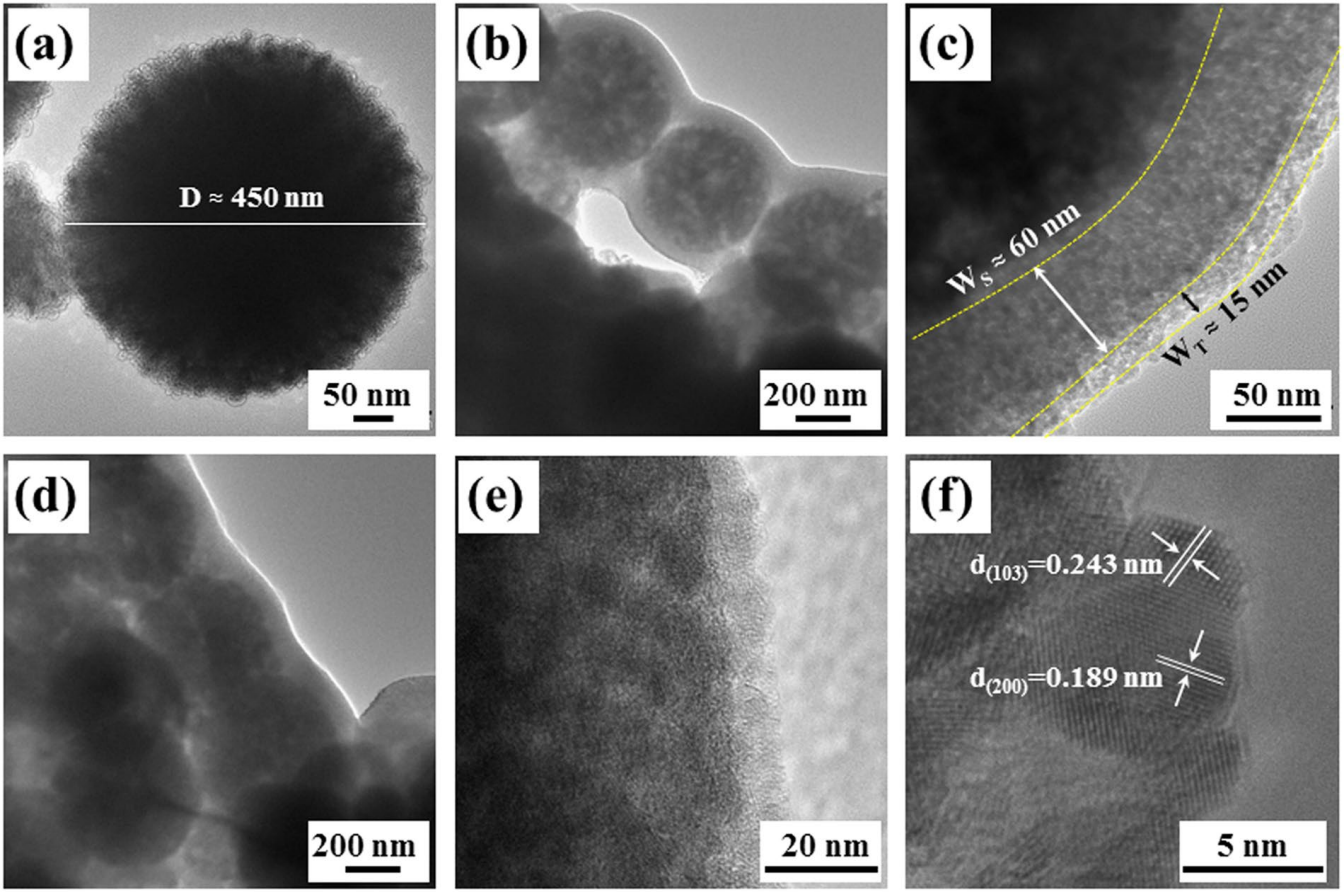
Typical HRTEM images of different samples: (**a**) Fe_3_O_4_, (**b**,**c**) Fe_3_O_4_@SiO_2_@TiO_2_, (**d**–**f**) γ-Fe_2_O_3_@SiO_2_@TiO_2_.

In order to reveal the structural characteristics of corn-like Fe_3_O_4_@SiO_2_@TiO_2_ heterostructure, HRTEM images were done on Fe_3_O_4_ and Fe_3_O_4_@SiO_2_@TiO_2_ before and after annealing. As seen from Fig. 4(a), the diameter of Fe_3_O_4_ is about 450 nm, and Fe_3_O_4_ porous microspheres possess a hierarchical structure consisting of densely packed nanoparticles with sizes of about 20 nm. Such morphology is beneficial for the subsequent surface coating. In Fig. 4(b), the uniform SiO_2_ and TiO_2_ shells are coated on Fe_3_O_4_ spheres to form one-dimensional chain-like Fe_3_O_4_@SiO_2_@TiO_2_ structures, which act as basic units to assemble into corn-like heterostructures. As shown in Fig. 4(c), one can clearly distinguish SiO_2_ and TiO_2_ shells from Fe_3_O_4_ cores in the as-prepared Fe_3_O_4_@SiO_2_@TiO_2_ microspheres. The thickness of SiO_2_ and TiO_2_ shells are close to 60 nm and 15 nm, respectively. The 60 nm SiO_2_ shell can not only act as a coupling layer for TiO_2_ coating, but also preserve the photocatalytic activity of TiO_2_ by inhibiting the electron transfer between the magnetic core and TiO_2_ shell. After annealing, the chain-like structural unit and a thinner TiO_2_ shell can be clearly observed in Fig. 4(d) and (e). Furthermore, the (103) and (200) lattice fringes of anatase TiO_2_ shown in Fig. 4(f) indicates that the TiO_2_ shell has been well-crystallized. This is in agreement with the XRD results of γ-Fe_2_O_3_@SiO_2_@TiO_2_ (Fig. 1). The average grain size of TiO_2_ is about 5 nm.

### Magnetic properties

**Figure 5:**
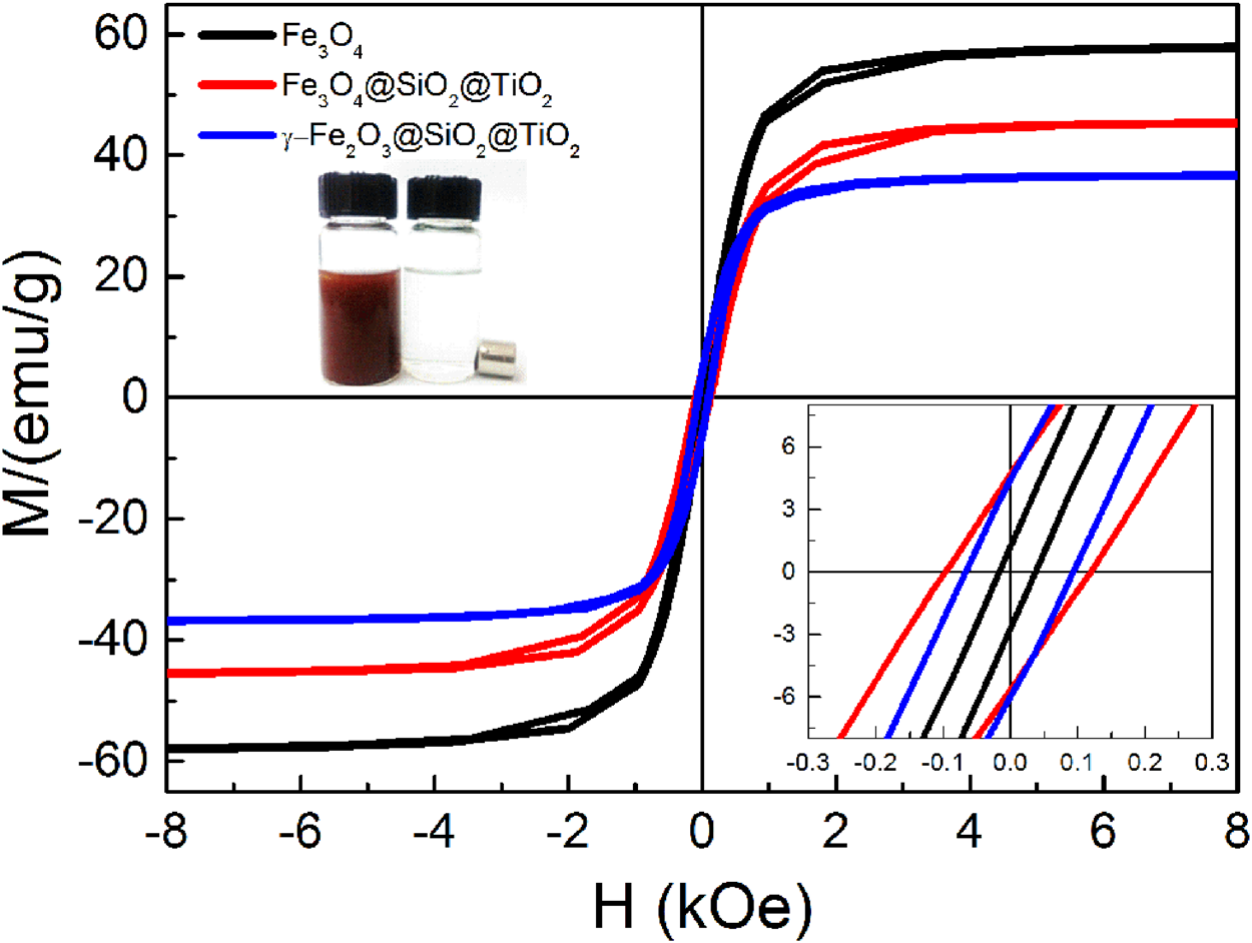
Magnetic hysteresis loops of Fe_3_O_4_ microspheres, corn-like Fe_3_O_4_@SiO_2_@TiO_2_ and γ-Fe_2_O_3_@SiO_2_@TiO_2_ heterostructures.

The room temperature magnetic properties of Fe_3_O_4_ microspheres, corn-like Fe_3_O_4_@SiO_2_@TiO_2_ and γ-Fe_2_O_3_@SiO_2_@TiO_2_ heterostructures are shown in Fig. 5. Fe_3_O_4_ microspheres have a saturation magnetization (*M*_*s*_) of about 58 emu/g. After coating of SiO_2_ and TiO_2_ shells, *M*_*s*_ of Fe_3_O_4_@SiO_2_@TiO_2_ heterostructure decreases to 45 emu/g due to the increased volume fraction of nonmagnetic materials. After annealing at 350 °C for 2 h in air, *M*_*s*_ of the γ-Fe_2_O_3_@SiO_2_@TiO_2_ heterostructure is further reduced to 37 emu/g, which is about 18% lower than that of Fe_3_O_4_@SiO_2_@TiO_2_. Such reduction in *M*_*s*_ is mainly due to the lower *M*_*s*_ of γ-Fe_2_O_3_ than that of Fe_3_O_4_. Nevertheless, the anisotropic corn-like heterostructures respond strongly to an external magnetic field, and can be efficiently extracted, as shown in the upper left inset. Such efficient separation is necessary for recyclable photocatalysts. For example, the collection time of 0.1 g γ-Fe_2_O_3_@SiO_2_@TiO_2_ in 20 ml ethanol is less than 10 seconds.

As seen from the lower right inset of Fig. 5, the isolated Fe_3_O_4_ microspheres show typical ferrimagnetic behavior but with insignificant remanent magnetization (*M*_*r*_). The low *M*_*r*_ is attributed to the multi-domain structure of the 450 nm-sized microspheres with small anisotropy. However, a higher *M*_*r*_ of about 5 emu/g is found for Fe_3_O_4_@SiO_2_@TiO_2_ which changes very little after annealing. The enhanced remanent magnetization of the heterostructure, on the other hand, is due to the formation of the anisotropic shape. As will be further discussed below, it is the magnetic dipole interactions between the microspheres lead to their self-assembly into chain-like structures and further corn-like heterostructure; while the chain formation in turn changes the magnetic behavior of the heterostructure. In the chain-like unit of the heterostructure, when the magnetization lies along the chain, there is a reduction of the stray field. This effective shape anisotropy results in the magnetic easy axis to lie along the chain. While this anisotropy is generally not large, it does increase both the remanent magnetization and coercivity of the assembly. Upon annealing, Fe_3_O_4_ is oxidized into γ-Fe_2_O_3_ with higher magnetocrystalline anisotropy, which further increases the coercivity of the γ-Fe_2_O_3_@SiO_2_@TiO_2_ corn-like assembly. The enhanced remanence is responsible for the enhanced magnetic recyclability under weak magnetic fields.

### Formation mechanism

**Figure 6:**
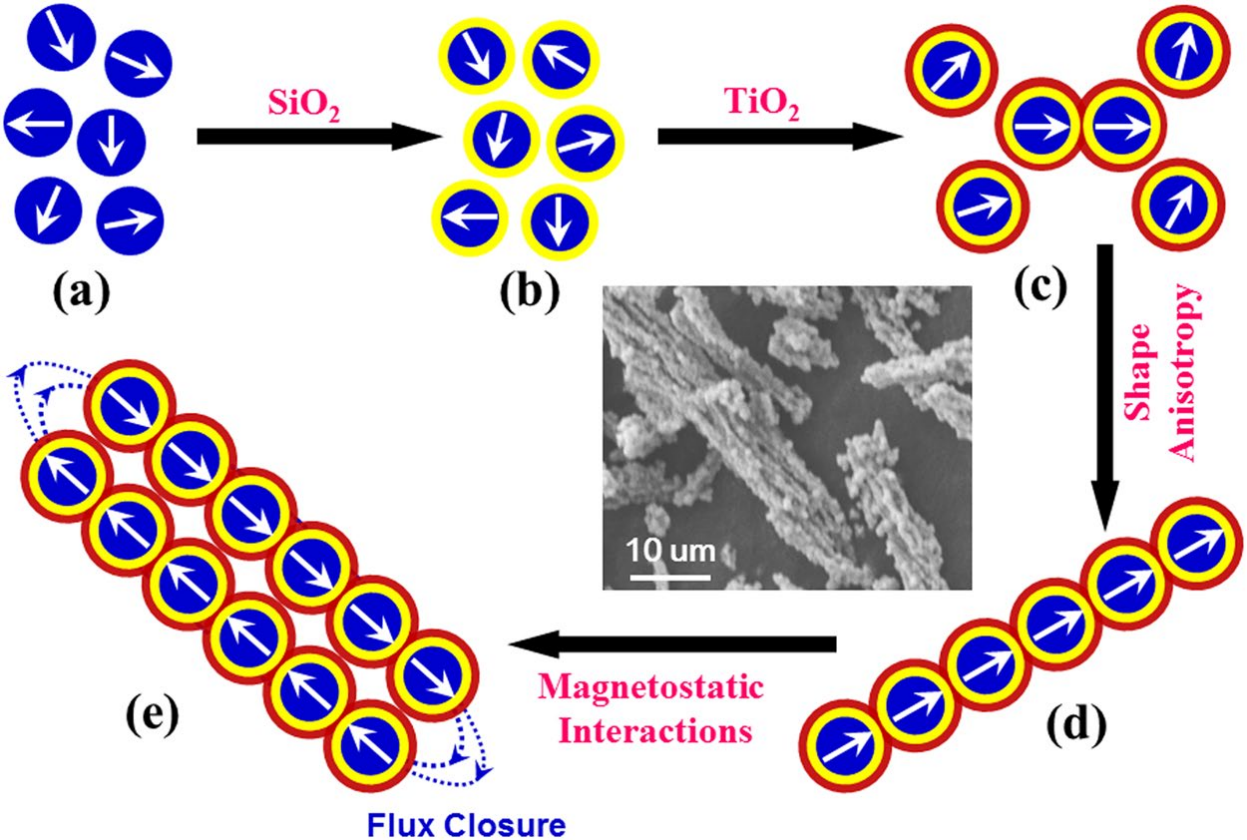
A schematic of the formation mechanism of Fe_3_O_4_@SiO_2_@TiO_2_ heterostructure.

It is interesting to note that the corn-like heterostructure is formed only in the Fe_3_O_4_@SiO_2_@TiO_2_, while in neither the Fe_3_O_4_ microspheres nor the Fe_3_O_4_@SiO_2_ core-shell structures. The chain-like structure is the basic component of these corn-like heterostructures. The formation is primarily a consequence of the competition between the magnetic dipole interactions, which favor chain-formation, and Brownian motion, which tends to randomize the assembly. We propose the following formation mechanism, as shown schematically in Fig. 6. During the first stage of synthesis [Fig. 6(a)], each isolated Fe_3_O_4_ microspheres can be regarded as a magnetic dipole. The dipole-dipole interactions between the microspheres tend to arrange the particles head-to-tail, forming a linear chain. However, the dipole moment in each microsphere is relatively small, due to the multidomain configuration. The random Brownian motion thus disrupts the chain formation. Moreover, the NaPAA ligand exerts steric forces, increasing the spacing between Fe_3_O_4_ microspheres, further reducing the dipole interactions. Therefore, Fe_3_O_4_ microspheres are dispersed as isolated entities at this stage. After SiO_2_ coating, the core/shell Fe_3_O_4_@SiO_2_ still maintains good dispersibility due to the long-chain capping groups of TEOS precursor on the microspheres [Fig. 6(b)]^44^.

However, during the TiO_2_ coating, the TiO_2_ colloidal sol caused by the hydrolysis of TTIP fill in the voids between Fe_3_O_4_@SiO_2_ microspheres, acting as a binder to fuse the neighboring microspheres. Once a Fe_3_O_4_@SiO_2_ dimer is formed, the symmetry is broken, and the axis along the dimer defines the magnetic easy axis, as shown in Fig. 6(c). The magnetization now tends to align along the dimer axis, increasing the effective dipole moment of the dimer. It is now energetically more favorable to connect more particles head-to-tail along the long axis to form a linear chain [Fig. 6(d)]. Thus, the chain-formation is a self-assembly process induced by magnetic dipole interactions, and assisted by the fusing effect of the TiO_2_ shell. Klokkenburg *et al*. first directly observed this dipolar chain formation in zero fields in a ferrofluid containing the largest synthetic single-domain magnetite particles^45^. The dipole-dipole interactions induced the anisotropic features, favoring a head-to-tail orientation. Moreover, Zhang *et al*. found that the Fe_3_O_4_ nanoparticles can self-assemble into one-dimensional chains in a colloidal dispersion through magnetic dipolar interaction without the help of an applied magnetic field^46^. As more chains are nucleated in the solution, when they touch by random motion, they tend to align side-by-side. This is akin to two bar magnets sticking together by aligning their north- and south- poles in opposite directions for flux closure to minimize magnetostatic interactions [Fig. 6(e)]. The flux closure is in Co particles simulated by Chantrell *et al*.^47^ We postulate that with increase in the numbers of chains, they order into tight bundles, forming corn-like heterostructures as depicted in the SEM image of Fig. 6. Moreover, the formation of corn-like heterostructures may be aided by Van der Waals interactions between the chains, as was reported in Fe_3_C microfiber assemblies^48^.

### Photocatalytic and magnetic recovery properties

**Figure 7:**
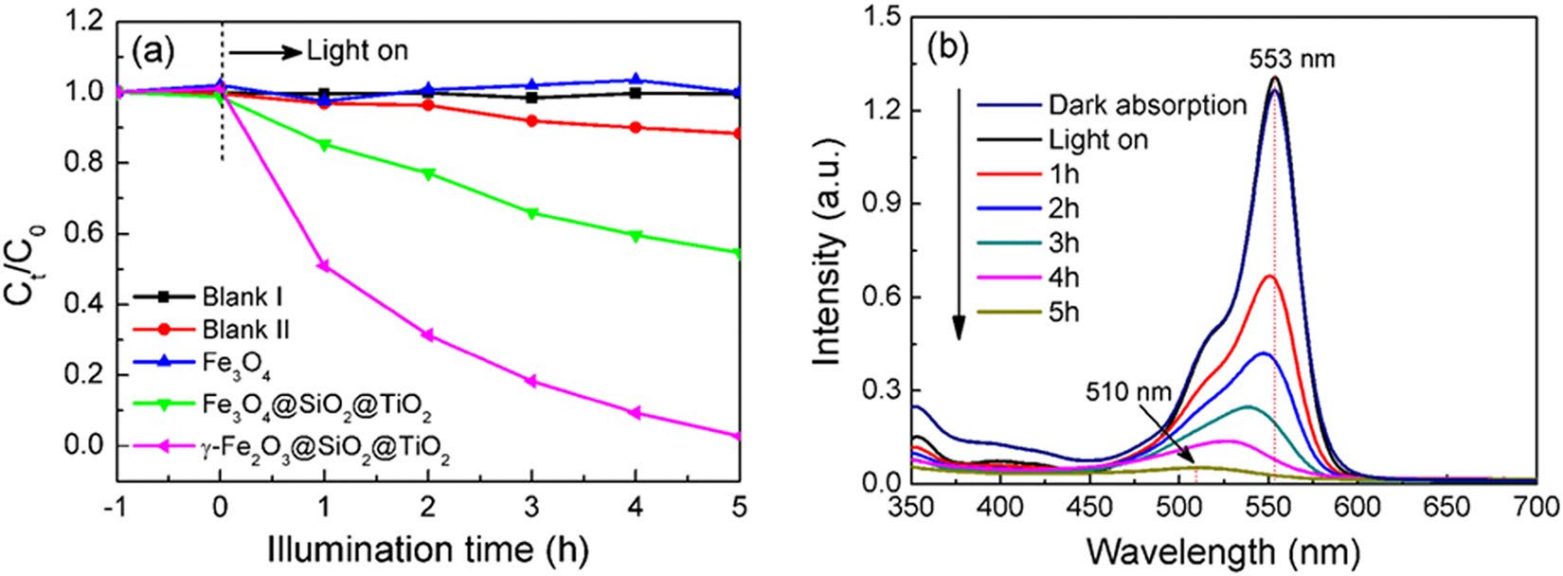
(**a**) Normalized concentration C_t_/C_0_ of RhB as a function of illumination time for Blank and different samples, (**b**) the UV-vis absorption spectra of RhB in the presence of γ-Fe_2_O_3_@SiO_2_@TiO_2_ sample as a function of illumination time.

The photocatalytic activity of Fe_3_O_4_, corn-like Fe_3_O_4_@SiO_2_@TiO_2_ and γ-Fe_2_O_3_@SiO_2_@TiO_2_ heterostructures were tested by measuring the photocatalytic degradation of RhB in water (10 mg/L) under the illumination of a Xe lamp (300 W). In order to ensure the reliability of the experiments, two control experiments, namely Blank I with catalysts only without light illumination (dark adsorption) and Blank II with light illumination only without catalysts (pure photolysis), were also performed. Figure 7(a) shows the normalized concentration of RhB (C_t_/C_0_) as a function of irradiation time for different photocatalyst and blank samples, in which C_t_ and C_0_ denotes the concentration of RhB aqueous solution at the irradiation time of t and t = 0 h, respectively. It can be seen that there is no measurable RhB degradation up to 5 h under light irradiation with Fe_3_O_4_ alone. However, RhB can be degraded rapidly in the presence of Fe_3_O_4_@SiO_2_@TiO_2_ and γ-Fe_2_O_3_@SiO_2_@TiO_2_ catalysts under light illumination. The normalized concentration C_t_/C_0_ reaches to nearly zero with the illumination time of 5 h for γ-Fe_2_O_3_@SiO_2_@TiO_2_, while it is only 0.55 for Fe_3_O_4_@SiO_2_@TiO_2_. It has been reported that the decrease in the bulk defects of TiO_2_ can enhance the separation of photogenerated electrons and holes, which results in improved photocatalytic activity. Kong *et al*. have also found that TiO_2_ synthesized at 120 °C exhibits the lower photocatalytic efficiency than the one prepared at 180 °C and calcined at 480 °C for 3 h, which is attributed to the recombination of most photogenerated charge carriers in the bulk defects for the former^49^. Moreover, Guo *et al*. have shown that the photocatalytic activity can be enhanced in ZnO tetrapods with less nonradiative defects^50^. Here the lower photocatalytic efficiency of Fe_3_O_4_@SiO_2_@TiO_2_ is attributed to the amorphous TiO_2_ shell. Amorphous TiO_2_ has high concentration of bulk defects, which may act as charge trapping centers, preventing the photo-generated carriers to be used for reactions. After annealing, TiO_2_ in the assembly is converted into highly crystalline anatase structure with low concentration of bulk defects, which possesses high photocatalytic activity. Thus the corn-like γ-Fe_2_O_3_@SiO_2_@TiO_2_ heterostructure demonstrates the highest degradation rate for RhB dye.

Figure 7(b) shows the corresponding evolution of UV-vis spectra for RhB as a function of time for γ-Fe_2_O_3_@SiO_2_@TiO_2_. The relative concentration of RhB (C_t_/C_0_) is extracted from the integrated UV-vis peak intensity. The decrease and shift in the maximum of the absorption peak suggest the reduction of the chromophoric group and thus the degradation of RhB molecules. As shown in Fig. 7(b), the maximum absorbance of RhB shifts gradually from 553 nm to 510 nm, and it remarkably fade away at the illumination time of 5 h. The final product has an absorption peak at 510 nm, which can be identified as an incompletely N-deethylated outcome of RhB, N-ethyl rhodamine (MER). Such a process indicates that RhB has been degraded in the presence of γ-Fe_2_O_3_@SiO_2_@TiO_2_ sample. The similar photodegradation process of RhB by TiO_2_ has been studied in detail earlier^51, 52^.

**Figure 8:**
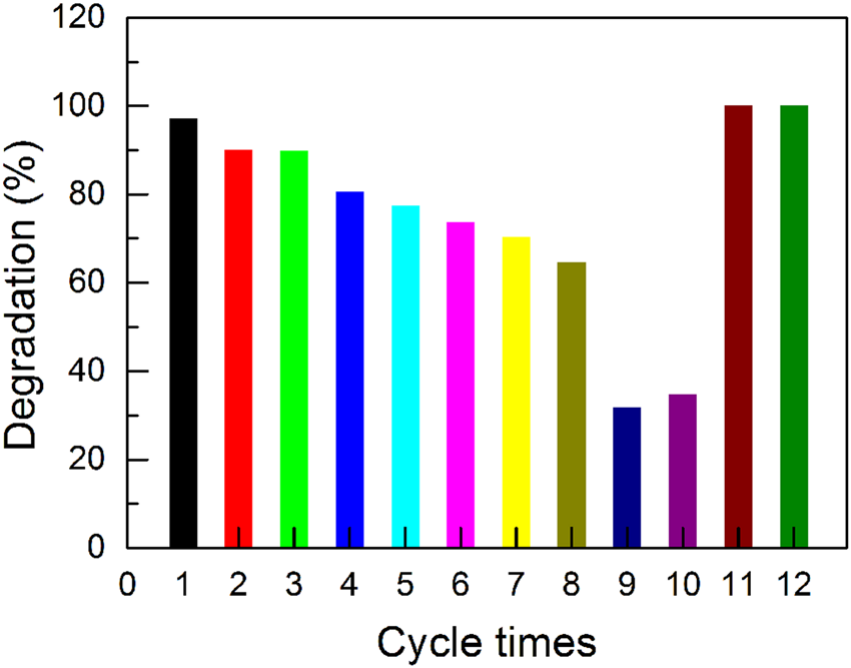
Cyclic tests of RhB degradation experiments with γ-Fe_2_O_3_@SiO_2_@TiO_2_ as the photocatalyst.

To assess the magnetic recyclability and photoactivity after recycling of the γ-Fe_2_O_3_@SiO_2_@TiO_2_ catalysts, the above experiment was performed, after which the catalysts were recycled by a magnet and re-used for the same experiment. This procedure was repeated for ten times. The percentage of degradation of RhB (at 5 h) after each experiment was calculated using (1 - C_t_/C_0_) × 100%. As shown in Fig. 8, a degradation percentage of 65% is retained after eight cycles, with an average loss of ~5% activity after each cycle. There is a large drop of the activity at the 9^th^ and 10^th^ cycles. We attribute the reduced activity to the adsorbed by-product from RhB photodegradation, which blocks the active sites of TiO_2_. We thus postulate that the photoactivity can be recovered by heating to remove the adsorbates. To ensure dispersibility of the catalysts, and prevent aggregation (reduces active surface area) and oxidation of γ-Fe_2_O_3_ to antiferromagnetic α-Fe_2_O_3_, the temperature should be as low as possible. We found that moderate heat treatment at 200 °C for 30 min in air is the optimal condition for regeneration of the photocatalysts. As can be seen in Fig. 8, after the recycled γ-Fe_2_O_3_@SiO_2_@TiO_2_ sample was subjected to the treatment, 100% RhB degradation was achieved at 5 h again, suggesting that the photocatalytic activity was fully recovered. The photocatalytic activity does not decrease after repeated regeneration (11^th^ and 12^th^ cycles in Fig. 8), demonstrating the reusability and durability of the catalysts.

## Conclusions

In summary, we have successfully synthesized anisotropic corn-like γ-Fe_2_O_3_@SiO_2_@TiO_2_ core/shell heterostructures by modified solvothermal and sol-gel methods. The formation mechanism of the hierarchical heterostructures is attributed to a self-assembly process induced by magnetic dipole interactions assisted by fusing effect of TiO_2_. The magnetic remanence is enhanced in the chain-like structure, facilitating magnetic recovery. The assembly demonstrates good photocatalytic activity and magnetic recyclability. While the catalytic activity decreases after each cycle due to adsorption of contaminants, it can be completely recovered by moderate heating. Therefore, our material is a robust and durable photocatalyst with excellent magnetic recyclability, and does not suffer from reduced surface area in supported catalysts.

## Methods

### Synthesis of Fe_3_O_4_ microspheres

All the reagents were analytical grade without further purification. Deionized water was used for all synthesis and post-treatment processes. Fe_3_O_4_ microspheres were synthesized by a modified solvothermal method^53^. Ferric chloride (FeCl_3_, 0.4 g) was dissolved in ethylene glycol (EG, 20 mL) to form a clear solution, and then sodium acetate (NaAc, 1.8 g) and sodium polyacrylate (NaPAA, 0.07 g) were added into it to obtain a mixture. The mixture was stirred vigorously for 30 min and then sealed in a Teflon-lined stainless-steel autoclave (20 mL capacity). The autoclave was maintained at 200 °C for 12 h, and allowed to cool to room temperature naturally. Fe_3_O_4_ microspheres were obtained. They were rinsed with ethanol, and then suspended in ethanol for later use. In addition, polyethylene glycol (PEG) and ethylene diamine (ED) were also tried as surfactants to control the morphology of Fe_3_O_4_.

### Synthesis of anisotropic, corn-like γ-Fe_2_O_3_@SiO_2_@TiO_2_ heterostructure

To obtain the anisotropic γ-Fe_2_O_3_@SiO_2_@TiO_2_ heterostructure, a SiO_2_ coupling layer was first coated on Fe_3_O_4_ by a sol-gel process^44^. Fe_3_O_4_ solution was maintained in a water bath at 40 °C, and then ammonia (25%, 89.5 mL), water (75 mL), and tetraethyl orthosilicate (TEOS, 1.5 mL) were added sequentially into the above Fe_3_O_4_ solution. The mixture was stirred vigorously for 2 h and followed by sonication for 1 h. Thus, Fe_3_O_4_ microspheres with a SiO_2_ shell can be obtained. The microspheres were rinsed with ethanol, and then resuspended in ethanol. This suspension was heated up to 60 °C for 12 h to strengthen the Si-O-Fe cross-linking on the surface of the Fe_3_O_4_ microspheres. The Fe_3_O_4_@SiO_2_ magnetic microspheres were then flocculated by applying a magnetic field to allow convenient removal of the free silica.

For TiO_2_ shell coating, 0.15 mL water and 5 mL ethanol were added into the suspension of Fe_3_O_4_@SiO_2_ microspheres, and then subjected to vortex-mixing for 15 min. Subsequently, a solution of titanium (IV) isopropoxide (TTIP, 0.5 mL) in 10 mL ethanol was added into the mixture, and then stirred vigorously in a 70 °C water bath for 4 h. The mixture was then sealed in an autoclave, heated at 120 °C for 5 h, and cooled to room temperature. The product was then rinsed and dried. To improve the crystallinity of TiO_2_ for better photocatalytic properties, the Fe_3_O_4_@SiO_2_@TiO_2_ heterostructures were annealed at 350 °C for 2 h in air. Upon annealing, TiO_2_ crystalized and Fe_3_O_4_ was oxidized into γ-Fe_2_O_3_, and as a result, the color turned from black for Fe_3_O_4_@SiO_2_@TiO_2_ to brown-red for γ-Fe_2_O_3_@SiO_2_@TiO_2_ heterostructures.

### Photocatalytic activity measurement

The photocatalytic activity of the samples was evaluated by the degradation of Rhodamine B (RhB) under the irradiation of a Xe lamp (300 W). 100 mL RhB aqueous solution with a concentration of 10 mg/L was mixed with 0.1 g catalysts in an optically matched Pyrex vessel. The reactor is tightly sealed and water cooled to a temperature of 25 °C to avoid heating effects under irradiation. The distance between the lamp and RhB solution is 10 cm. Before the irradiation, the suspension was stirred in dark condition until an adsorption-desorption equilibrium was established. Samples of the solution were taken out from the reactor every one hour, and the concentration of RhB was analyzed by UV-vis spectra. At the end of each photocatalytic cycle, the photocatalysts were magnetically collected, and then rinsed with water for the next cycle use.

### Characterizations

X-ray diffraction (XRD) patterns were recorded with a powder X-ray diffraction instrument with Cu Kα radiation (λ = 1.54 Å). The elements and valence states were characterized using X-ray photoelectron spectroscopy (XPS). The morphologies were observed on a field emission scanning electron microscopy (FESEM) and high-resolution transmission electron microscopy (HRTEM). Magnetic properties were carried out in a vibrating sample magnetometer (VSM) at room temperature.
